# Quality of Life in Women with Defecatory Dysfunctions: Systematic Review of Questionnaires Validated in the Portuguese Language

**DOI:** 10.1055/s-0039-1678592

**Published:** 2019-03-01

**Authors:** José Ananias Vasconcelos Neto, Camila Teixeira Moreira Vasconcelos, Sara Arcanjo Lino Karbage, Hérdeny Di Cárlly de Almeida Rocha Farias, Stéffany Gadelha de Macêdo Machado, Dayana Maia Saboia

**Affiliations:** 1 Department of Urogynecology, Hospital Geral de Fortaleza, Fortaleza, CE, Brazil; 2 Department of Nursing, Universidade Federal do Ceará, Fortaleza, CE, Brazil; 3 Department of Urogynecology, Maternidade Escola Assis Chateaubriand, Fortaleza, CE, Brazil; 4 Department of Urogynecology, Hospital Geral de Fortaleza, Fortaleza, CE, Brazil; 5 Department of Medicine, Universidade Estadual do Ceará, Fortaleza, CE, Brazil

**Keywords:** fecal incontinence, quality of life, validation studies, surveys and questionnaires, review, incontinência fecal, qualidade de vida, estudos de validação, inquéritos e questionários, revisão

## Abstract

**Objective** To identify the quality of life (QoL) assessment instruments related to the health of women with fecal incontinence (FI) or anal incontinence (AI).

**Data Sources** Systematic review conducted in the Virtual Health Library (VHL), PubMed and Cochrane Library databases. The descriptors used were: *Questionnaire*, *Questionnaires*, *Quality of life*, *validation*, *validation Studies*, *anal incontinence*, *fecal incontinence* and *constipation*. The search was performed between December 26, 2017 and the beginning of January 2018. The limits used were female gender.

**Selection of Studies** Initially, 5,143 articles were obtained in the search. The articles of validation for Portuguese of questionnaires for the evaluation of the impact of FI/AI on the QoL of women were considered eligible.

**Data Collection** The article search was conducted according to the Preferred Reporting Items for Systematic Reviews and Meta-Analyzes (PRISMA) guidelines.

**Data Synthesis** Of the 5,143 articles, only 2 fulfilled the inclusion and exclusion criteria: Fecal Incontinence Quality of Life (FIQL) and the Wexner scale (WS). The FIQL evaluates the QoL related to FI, not covering flatus incontinence. The WS assesses flatus incontinence and the severity of the AI. The WS obtained an interclass correlation coefficient (ICC) of 0.932 and a Cronbach α coefficient > 0.90. The FIQL obtained intraexaminer and interexaminer reproducibility ranging from 0.929 to 0.957 and from 0.944 to 0.969, respectively.

**Conclusions** The WS and the FIQL have satisfactory reliability and validity for use during gynecological consultations.

## Abstract (pt)

**Resumo:**

**Objetivo** Identificar os instrumentos de avaliação da qualidade de vida (QV) relacionados à saúde de mulheres com incontinência fecal (IF) ou incontinência anal (IA).

**Fontes dos dados** Revisão sistemática nas bases de dados da BVS, PubMed e Biblioteca Cochrane. Os descritores usados foram: *Questionnaire, Questionnaires, Quality of life, validation, validation Studies, anal incontinence, fecal incontinence* e *constipation*. A pesquisa foi realizada entre 26 de dezembro de 2017 até o início de janeiro de 2018. Os limites utilizados foram sexo feminino.

**Seleção dos estudos** Inicialmente, 5.143 artigos foram obtidos na pesquisa. Os artigos de validação para o português de questionários de avaliação do impacto da IF/IA na QV das mulheres foram considerados elegíveis.

**Coleta de dados** A busca de artigos foi conduzida de acordo com as diretrizes do Principais Itens para Relatar Revisões Sistemáticas e Meta-análises (PRISMA, na sigla em inglês).

**Sínteses dos dados** Dos 5.143 artigos, apenas 2 preencheram os critérios de inclusão e exclusão: Qualidade de Vida em Incontinência Fecal (FIQL, na sigla em inglês) e Escala de Wexner (WS, na sigla em inglês). O FIQL avalia a QV relacionada à FI, não abrangendo a incontinência de gases. O WS avalia a incontinência dos flatos e a gravidade da IA. O WS obteve um coeficiente de correlação interclasse (ICC, na sigla em inglês) de 0,932 e alfa de Cronbach > 0,90. O FIQL obteve reprodutibilidade intraexaminador e interexaminador variando de 0,929 a 0,957 e de 0,944 a 0,969, respectivamente.

**Conclusões** O WS e o FIQL têm confiabilidade e validade satisfatória para uso durante consultas ginecológicas.

## Introduction

Anal incontinence (AI) is part of a spectrum of defecatory function disorders that also includes incomplete bowel movement, urgency, change in bowel frequency, painful defecation, and constipation. It is the most debilitating symptom of pelvic floor dysfunction (PFD), a clinical entity that encompasses fecal incontinence (FI), urinary incontinence (UI), and pelvic organ prolapse (POP), with a greater psychosocial impact on women.^1^ ^2^

In a study in which 226 women with FI were evaluated, it was observed that 35.6% of the patients had moderate to severe quality of life (QoL) impairment.^3^ When evaluating women with PFD, 54.6% had defecation disorders, of which 41.4% corresponded to AI.^4^

It is estimated that the prevalence of AI in the general population can vary between 0.4 and 18%, and may reach a rate of 20% in the population > 40 years old. It is likely that these values are even greater due to the underreporting of the disease.^3^ ^5^ In Brazil, 1 study found a prevalence of 7% in the adult population, with similar rates in both sexes. Regarding the type of loss, a prevalence of 3.5% was observed for fecal losses (3.1% for men and 4.2% for women), and of 4% for gases.^6^

Currently, QoL assessment is used in the clinical practice, aiming to verify the impact of the disease in the life of the patient and to help in choosing the best treatment. The QoL assessment has been discussed and highlighted as an ethical, professional, and economical indicator to improve diagnosis and promote treatment efficiency. The results of the interventions have also been evaluated considering the perception of the patients of their well-being and expectations, including physical, social, emotional, and occupational aspects.^7^

Considering this scenario, the aim of the present study was to identify and analyze QoL questionnaires related to FI/AI and constipation validated for the Portuguese language, seeking to contribute to the dissemination of these instruments in the scientific scenario and in the clinical practice and, thus, to improve knowledge and healthcare.

## Methods

A systematic review was performed in the following databases: PubMed, Virtual Health Library (VHL) and Cochrane Library. The descriptors used were: *Questionnaire, Questionnaires, Quality of life, validation, validation studies, anal incontinence, fecal incontinence* and *constipation* (Table 1).

**Table 1:** PICO strategy

	Definition	Descriptors	Limits
**Patient**	Brazilian women with anal incontinence or constipation	*Anal incontinence* OR *Fecal Incontinence* OR *Constipation*	Women
**AND**			
**Intervention**	Anal incontinence or constipation questionnaire	*Questionnaire* *OR* *Questionnaires*	
***OR***			
**Comparison**	No other questionnaire or other evaluation questionnaire		
**Outcome**	Validation, reliability	*Validation* *OR* *Validation Studies* *OR* *Quality of life*	

In this search, which was performed between December 26, 2017 and the beginning of January 2018, no filters were used. The article search was conducted according to the Preferred Reporting Items for Systematic Reviews and Meta-Analyzes (PRISMA) guidelines.

The articles that covered validation studies for Portuguese of QoL questionnaires related to FI/AI and constipation in women were considered eligible. Articles that did not provide the full text, duplicate articles, articles of validation of questionnaires modified for use in specific populations (patients with rectal cancer) or in patients who were opioid users were excluded. The selection of the studies was performed in three stages. In the first stage, two reviewers proceeded to read the titles of the works found. In the second stage, the abstracts of the selected articles were read. Finally, the selected articles were read in full and evaluated for the following variables:

- Identification of the publication: authorship, year of publication, journal;
- Description of the questionnaire: name of the instrument, number of questions, time spent to apply the questionnaire, and form of application of the instrument;
- Validation of the questionnaire: target population, sample, age, reliability, validity, and limitations.^8^ ^9^

## Results

**Fig. 1:**
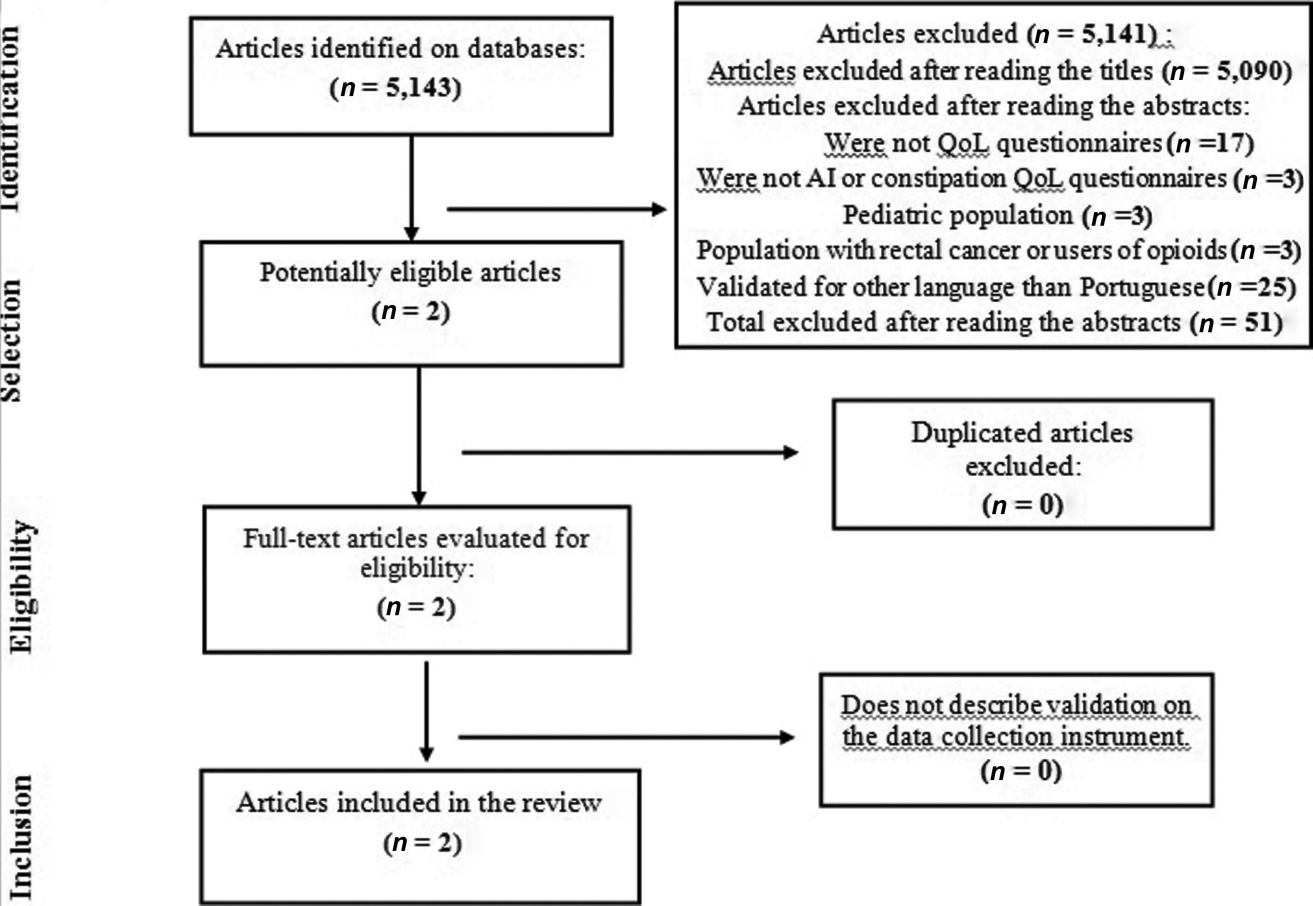
Design of the PubMed, VHL and Cochrane search for articles of validation for Portuguese of quality of life questionnaires for anal incontinence and constipation.

Initially, 5,143 articles were obtained in the search. After reading the titles, 5,090 articles were excluded because they did not meet the inclusion criteria, with 53 articles being selected (Fig. 1).

Of these, 51 were excluded after reading the abstracts according to the exclusion criteria: 17 did not assess the impact on the QoL; 3 were not FI/AI specific QoL questionnaires; 3 encompassed the pediatric population; 3 validated specific questionnaires for the population with rectal cancer or opioid users, and 25 were validated for languages other than Portuguese. Two articles, therefore, remained to be read in full. The references of the two selected articles were evaluated; however, no study was added. Finally, two validation articles for Portuguese were obtained: Wexner Scale (WS) and Fecal Incontinence Quality of Life (FIQL),^8^ ^9^ the main characteristics of which are summarized in Table 2 and Table 3.

**Table 2:** Structure of the selected validation studies: Wexner Scale and Fecal Incontinence Quality of Life questionnaire

Authors	Questionnaire	Number of questions	Items/ Domains	Questions	Score variation	Time	Application
**Fonseca et al (2016)** 8	Wexner Scale	5 questions	Solid Liquid Gas Use of protectors Alteration of the lifestyle	1 2 3 4 5	0–4 0–4 0–4 0–4 0–4	Not found	Interview
**Yusuf et al (2004)** 9	Fecal incontinence quality of life (FIQL)	4 questions (Total of 29 items): Q1: 1 item Q2: 13 items Q3: 14 items Q4: 1 item	Lifestyle	10 questions: Q2 (items a, b, c, d, e, g, h) Q3 (items b, l, m)	1–4 1–4 1–4 1–4 1–5 1–4 1–6 1–4 1–4	Average time: 13 minutes	Interview
			Behavior	9 questions: Q2 (items f, I, j, k, m) Q3 (items c, h, j, n)			
			Depression	7 questions: Q1 Q3 (items d, f, g, i, k); Q4			
			Embarrassment	3 questions: Q2 (Item l) Q3 (items a, e)			

Abbreviations: Q1, question 1; Q2, question 2; Q3, question 3; Q4, question 4.

**Table 3:** Population and validation details of the selected validation studies: Wexner Scale and Fecal Incontinence Quality of Life questionnaire

Authors	Target population	Sample	Age (mean)
**Fonseca et al** 8	Patients from the urogynecology outpatient clinic	20 patients with AI for the cultural translation and adaptation 50 patients with AI for the validation	57.5 years old
**Yusuf et al** 9	Patients with AI from the physiology of the colon, rectum, and anus outpatient clinic	40 patients with AI for the cultural adaptation; 50 patients with AI for the reproducibility and construct validity; 30 patients with constipation and 30 healthy patients for the discriminative validity Total: 150 patients	52.8 years old
**Authors**	**Reliability**	**Validity**	**Limitation**
**Fonseca et al** 8	Cronbachs α = 0.932	Convergent validity and discriminant validity	The authors did not verify whether the order of application of the face-to-face interview/telephone interview affected the results. Lack of evidence of sensitivity to change. 7.2% of the patients were illiterate (could be considered a bias in favor of successful results.)
**Yusuf et al** 9	Intraexaminer and interexaminer reproducibility ranged from 0.929 to 0.957 and from 0.944 to 0.969, respectively (ICC)	Construct validity and discriminatory validity	In 22% of the cases, there were no results of the AII corresponding to the quality of life index (10% with a slight impact on the quality of life despite high AI/12% with moderate or mild AI), with great repercussions on QoL). 26% of the population was composed by men.

Abbreviations: AI, anal incontinence; AII, anal incontinence index; ICC, intraclass correlation coefficient; QoL, quality of life.

### Wexner Scale (WS) 

### Construction of the Questionnaire

At the beginning of the validation process of the WS for Brazilian Portuguese, experienced native speakers and professional translators provided the first two independent translations, developing a single combined version. The first version in Portuguese, back-translated to the English language by two specialists, and the results were compared with the original instrument in American English. There was no discrepancy between the versions. Next, during a meeting with coloproctologists, urogynecologists and physiotherapists, the Brazilian Portuguese version of the WS was created.

From this, patients (70) with symptoms of AI were selected from the urogynecology outpatient clinic of a university hospital. The study took the form of an interview conducted by the researcher because many of the patients could not read or write. Out of the total sample, 5 patients were illiterate, 53 had completed elementary school, 10 had completed high school, and 1 had attended further education.

The first version of the questionnaire was tested in 20 patients with AI. To perform this test, the response “I do not understand” was included at the end of each question of the scale. The questions to which this type of response was > 15% were considered difficult to understand by this population and, therefore, were modified. Thus, the final version of the Brazilian Portuguese scale was developed.

### Description of the Questionnaire

The questionnaire consists of five questions: three about AI (gas, liquid and solid), one about the loss mechanism (use of pads), and one lifestyle question (change). The score used was the same as that of the original questionnaire, and the interviewees were instructed to evaluate the frequency of fecal loss, the frequency of the use of pads, and the frequency of lifestyle changes through the use of quantifiers (0 = never, 1 = rarely, 2 = sometimes, 3 = usually, 4 = always). The final score was obtained through the sum of the points. Higher scores indicate greater severity of the AI. The total score in the instrument ranges from 0 (no incontinence) to 20 (complete incontinence).

### Validation of the Questionnaire

After the process of translation and cultural adaptation, the WS questionnaire, already in the final Brazilian Portuguese version, along with the FIQL questionnaire, were applied in another 50 women suffering from AI. The convergent validity was assessed by comparing the data from the first WS interview with the FIQL using the Spearman correlation test.

The reproducibility (retest reliability) was evaluated over a 2-week interval, with the reapplication of the questionnaire by telephone. A total of 49 women responded to the questions. The answers of the two completed questionnaires were then analyzed.

The retest reliability was assessed using the intraclass correlation coefficient (ICC). The internal consistency was assessed using the Cronbach α coefficient (Table 4).

**Table 4:** Internal validity of the Portuguese version of the Wexner Scale (WS) (Cronbach αcoefficient) and intraclass correlation coefficients of the validation of the Fecal Incontinence Quality of Life (FIQL) questionnaire for Portuguese

Variable (WS)	Cronbach α coefficient	
**Solid**	0.799	
**Liquid**	0.768	
**Gas**	0.765	
**Use of protectors**	0.896	
**Alteration of the lifestyle**	0.865	
**Total score**	**0.932**	
**Variables (FIQL)**	Intraexaminer ICC	Interexaminer ICC
**Lifestyle**	0.934	0.944
**Behavior**	0.938	0.973
**Depression**	0.957	0.957
**Embarrassment**	0.929	0.969

Abbreviation: ICC, intraclass correlation coefficient.

Source: Fonseca et al (2016)^8^ and Yusuf et al (2004).^9^

## Description of the Study Population

The study population had a mean age of 57.5 years old, with the majority (77.1%) in the postmenopausal period. The mean body mass index (BMI) was 28.7. The mean number of pregnancies was 4 (0–15), and the mean number of vaginal deliveries was 3 (0–9). Only 10% of the patients were smokers, with no specification regarding the chronicity of the habit.

Concerning intestinal habits, 40 participants reported a regular habit, 19 reported constipation, and 11 reported diarrhea. Only 34% sought treatment for AI, and 28% reported having had been investigated by a physician about these symptoms.

The majority of these women (84.3%) had concomitant urinary incontinence (UI). According to the patients self-reported symptoms, 23.3% presented stress UI, 20% urge incontinence, and 51.7% mixed incontinence.

## Fecal Incontinence Quality of Life (FIQL)

### Construction of the Questionnaire

The original FIQL questionnaire was translated into Brazilian Portuguese by two English teachers. The versions were compared by a multidisciplinary group composed of two physicians, one nurse, and two psychologists; and, through consensus, the first version of the questionnaire in Brazilian Portuguese was produced. This version was translated into English by two professors of American nationality. This time, the multidisciplinary group evaluated all the versions, producing the second version of the questionnaire in Brazilian Portuguese, which was used for the process of cultural adaptation.

The questionnaire was then applied in a group of 20 patients with AI, randomly selected at the coloproctology outpatient clinic of a university hospital. For each question in the second version of the questionnaire, the “not applicable” option was added to identify which issues would not be culturally compatible or not understood by the Brazilian population. The questions that presented > 15% of “not applicable” responses were selected and rewritten by the multidisciplinary group, trying to preserve the original concept.

Subsequently, another 20 incontinent patients were selected, in whom the third version of the questionnaire was applied, observing that no question presented a “not applicable” response > 15%. Therefore, the cultural adaptation was considered complete, this being the final version of the questionnaire. The mean duration of each application was of 13 minutes.

### Description of the Questionnaire

The FIQL is a specific instrument to evaluate the QoL in relation to FI. It is composed of 29 questions divided into 4 domains: lifestyle, behavior, depression, and embarrassment. The questions are scored from 1 to 4, except for questions 1 and 4, which range from 1 to 5 and from 1 to 6, respectively. Lower scores indicate a worse QoL.

### Validation of the Questionnaire

For the performance of this stage, 50 patients with AI were selected. The reproducibility between different examiners was assessed through interviews conducted by examiners A and B on the same day, with the same patient, with a 30-minute interval between the interviews. Examiner A re-evaluated all of the patients after a period of between 7 and 10 days to compare the results obtained by the same examiner at different times. The results obtained for intra- and interexaminer reproducibility showed a significant agreement in all domains of the questionnaire. The ICCs of the evaluated domains are presented in Table 3.

In the construct validity analysis, incontinent patients responded to the Anal Incontinence Index (AII) and to the generic questionnaire known as Short Form 36 (SF-36). The results obtained were correlated with those of the FIQL. A significant correlation was found between all FIQL and SF-36 domains (coefficient 0.754 to 0.556, *p *< 0.01), except for the pain domain (coefficient = 0.103, *p* = NS).

In the evaluation of the discriminative validity, the FIQL was applied in 30 patients with intestinal constipation and in 30 healthy volunteers. It was observed that the QoL of the AI patients was compromised in all the domains covered by the FIQL when compared with the healthy volunteers and with patients with intestinal constipation.

In the statistical analysis, the ICC, analysis of variance (ANOVA), the Pearson coefficient, and the Student *t*-test were used. The significance level established was of 0.05. Patients with more severe AI (higher indexes) had worse QoL indexes (lower values), leading to the conclusion that there was an inverse correlation between the total values of the FIQL and of the AII. In 11 cases (22%), no correlation was observed between the FIQL values and those of the AII.

#### Description of the Study Population

The population was composed of incontinent patients, the majority being female (74%). The mean age was 52.8 years old (range: 15–75 years old). Regarding the level of education, the majority (88%) was literate. Concerning the work situation, 40 participants (80%) were employed, with 12% of those unemployed attributing the cause of unemployment to the incontinence. The mean duration of the AI was of 10.2 years.

## Discussion

Anal incontinence is a pathology that has a significant impact on the QoL. However, the number of instruments for its evaluation is limited.^10^ ^11^ ^12^ The first specific instrument for the assessment of AI was the FIQL in its original version, which was prepared in 2000 and was proposed by Rockwood et al,^13^ Other instruments in the literature are: the Wexner scale (WS), the Manchester Health Questionnaire (MHQ), the Modified Manchester Health Questionnaire (MMHQ), and the Rapid Assessment Faecal Incontinence Score (RAFIS).^8^ ^14^ ^15^ ^16^

Even fewer QoL instruments related to AI have been validated for Brazilian Portuguese. From the systematic review, only two instruments were found: the FIQL and the WS.^8^ ^9^

The FIQL is the most widely used instrument in the international literature, validated in several different languages: French, Italian, German, Spanish, Norwegian, Turkish, Chinese, Japanese, English, and Portuguese, while the WS has only been validated in Portuguese and in Turkish.^8^ ^17^ ^18^ ^19^ ^20^ ^21^ ^22^ ^23^ ^24^ ^25^ The FIQL evaluates the QoL of the FI patient without addressing the loss of gases, which differs from the WS. This fact is shown to be an advantageous aspect of the WS, since flatus incontinence is fairly common and is often the only manifestation of AI, which has an impact on the QoL, especially when associated with fecal loss. The addition of flatus loss to FI not only adds a negative psychological burden but can also have a significant overall impact on the general well-being of the patient.^12^

The WS consists of 5 items, 1 about lifestyle change, another about loss mechanisms, and 3 questions addressing incontinence (liquid, solid and gas), which makes its application quicker and simpler when compared with the FIQL, which has 29 items distributed in 4 domains: lifestyle, behavior, depression, and embarrassment. FIQL evaluates the QoL related to the FI patient in a more integrated way, analyzing different emotional aspects such as depression, shame, and sadness, as well as changes in daily life occurring due to the incontinence. However, it has the limitation of requiring a longer time for its application, which can make the use of the WS more attractive for research, as well as both for the researcher and for the patient, since it requires less time to be applied and is more objective, as well as being widely accepted by the scientific community.^8^ ^9^

Both validations included patients with AI, with the FIQL sample being larger than that of the WS. In addition, the validation of the FIQL included patients of both genders, predominantly women (74%), while that of the WS included only female patients. This data corroborates the fact that AI is more prevalent in females. Nelson et al (1995),^26^ in a study with the population of the community of an American city, reported that 2.2% of the general population had AI, of which 63% were women. The female gender is indicated as a risk factor for AI due to pregnancy and vaginal delivery.^6^ ^26^ ^27^

The mean age of the patients in both studies was > 50 years old (57.5 years old in the WS, and 52.8 years old in the FIQL). The study by Townsend et al (2013)^27^ showed that the prevalence of liquid or solid feces incontinence at least monthly increased from 9% in women aged between 62 and 64 years old to 17% in women aged between 85 and 87 years old. In a Brazilian study by Zaslavsky et al (2012),^28^ it was observed that people with AI have a significantly greater mean age than those without it, with the age of > 41 years old being significantly associated with the presence of AI.^27^ ^28^

According to the data obtained in the WS validation study, it was observed that 84.3% of the women had concomitant UI, which was not exposed in the validation of the FIQL.^8^ In 2014, a study that evaluated symptoms of PFD found that 23.21% of the women with UI had associated AI.^4^ In another study, it was concluded that UI has a strong coexistence with FI, with 63% of the women with FI reporting UI at least monthly compared with 45% of the women of the entire study population.^27^

The reproducibility of the WS was evaluated within a 2-week interval through the test-retest technique, using the ICC, which was 0.932, demonstrating a good reliability. This was also evaluated through the Cronbach α coefficient, which showed a high level of internal consistency (α > 0.90). The FIQL validation study used only the ICC in the reliability analysis, also showing a good reproducibility. This reveals that both questionnaires have good reliability and reproducibility.

To analyze the construct validity of the FIQL, the study participants also responded to the SF-36 questionnaire and to the AII. An inverse correlation was found between the total values of the FIQL and of the AII, showing that the severity of the AI compromised the QoL. In the comparison with the SF-36, a significant correlation was observed between all domains, except that of pain. On the other hand, WS used only one element for the construct validity, the FIQL, which revealed a proportionally inverse relationship between them.

The limitations of the validation study of the WS consisted of not verifying whether the order of administration of the face-to-face interview/telephone interview affected the results; not being able to recommend a different order to the one that was used; lack of evidence of sensitivity to change; and the fact that 7.2% of the patients were illiterate (which could be considered a bias in favor of successful results). During the validation of the FIQL, it was observed that 26% of the population was composed by men, and that in 22% of the cases there was no correspondence between the AI and the QoL (10% with a slight impact on the QoL despite the high AII values, and 12% with moderate or mild AI with a high impact on the QoL). This finding was related to the sociocultural factors inherent to the individual.^8^ ^9^

## Conclusion

The present study showed that the QoL of the Brazilian population affected by AI can be evaluated through two validated questionnaires (the WS and the FIQL) that are widely accepted and used by the scientific community. Both questionnaires demonstrated satisfactory reliability and validity, being reliable, consistent, and valid instruments for the assessment of QoL related to AI. The WS, being shorter, can be used in the screening process for the identification of patients with FI/AI symptomatology, while the FIQL may be reserved for follow-up treatment or for comparisons in international studies.
